# V3 Loop Sequence Space Analysis Suggests Different Evolutionary Patterns of CCR5- and CXCR4-Tropic HIV

**DOI:** 10.1371/journal.pone.0007387

**Published:** 2009-10-09

**Authors:** Katarzyna Bozek, Alexander Thielen, Saleta Sierra, Rolf Kaiser, Thomas Lengauer

**Affiliations:** 1 Max Planck Institute for Informatics, Saarbrücken, Germany; 2 Institute for Virology, University of Cologne, Cologne, Germany; New York University School of Medicine, United States of America

## Abstract

The V3 loop of human immunodeficiency virus type 1 (HIV-1) is critical for coreceptor binding and is the main determinant of which of the cellular coreceptors, CCR5 or CXCR4, the virus uses for cell entry. The aim of this study is to provide a large-scale data driven analysis of HIV-1 coreceptor usage with respect to the V3 loop evolution and to characterize CCR5- and CXCR4-tropic viral phenotypes previously studied in small- and medium-scale settings. We use different sequence similarity measures, phylogenetic and clustering methods in order to analyze the distribution in sequence space of roughly 1000 V3 loop sequences and their tropism phenotypes. This analysis affords a means of characterizing those sequences that are misclassified by several sequence-based coreceptor prediction methods, as well as predicting the coreceptor using the location of the sequence in sequence space and of relating this location to the CD4^+^ T-cell count of the patient. We support previous findings that the usage of CCR5 is correlated with relatively high sequence conservation whereas CXCR4-tropic viruses spread over larger regions in sequence space. The incorrectly predicted sequences are mostly located in regions in which their phenotype represents the minority or in close vicinity of regions dominated by the opposite phenotype. Nevertheless, the location of the sequence in sequence space can be used to improve the accuracy of the prediction of the coreceptor usage. Sequences from patients with high CD4^+^ T-cell counts are relatively highly conserved as compared to those of immunosuppressed patients. Our study thus supports hypotheses of an association of immune system depletion with an increase in V3 loop sequence variability and with the escape of the viral sequence to distant parts of the sequence space.

## Introduction

Host cell entry of HIV-1 is mediated by viral membrane-bound proteins [1]. The initial contact between the viral envelope glycoprotein gp120 and the cellular receptor CD4 is followed by a second interaction between gp120 and one of the cellular coreceptors: CCR5 or CXCR4 [2], [3]. It has been shown that viruses binding to CCR5 are almost exclusively present during the early asymptomatic stage of the infection whereas CXCR4-binding viruses may emerge in later phases of the infection and are associated with a CD4^+^ T-cell decline and progression towards AIDS [4]. The specificity of the virus to use one of the coreceptors is often termed tropism. Before the coreceptors were identified, two phenotypic variants were recognized according to the virus' ability of forming syncytia in MT-2 cells. Already at that time, syncytium-inducing (SI) and non-syncytium-inducing (NSI) viruses were observed to have a different impact on the disease progression in infected people [5]. There is a high correlation between CCR5-tropic and NSI viruses, on the one hand, and between CXCR4-tropic and SI viruses, on the other hand. The question whether the emergence of CXCR4 and SI virus is a cause of advanced progression towards CD4^+^ T-cell depletion and the rise of AIDS symptoms or appears as a result of these phenomena (or both), as well as the evolutionary reasons for the development of these variants remain largely unresolved.

The capacity of HIV-1 to use a specific coreceptor resides mainly in the sequence of the V3 loop of the viral envelope protein gp120. Current coreceptor prediction methods (e.g. 11/25 rule, WebPSSM, geno2pheno) [6], [7], [8] aim at revealing the relationship between V3 loop sequence and viral coreceptor usage. However, the overall reliability of sequence-based methods for coreceptor prediction is still limited [8].

In this work, we present the results of a comprehensive analysis of the viral V3 loop sequence space. Using different sequence distance measures and visualization methods we describe the arrangement of the sequences in sequence space. Our results reveal a relatively high conservation of CCR5-tropic and NSI strains as compared to more diverse CXCR4-tropic and SI strains evolving in an apparently unconstrained manner. On the one hand, we find that the arrangement of the sequences imparts one of the reasons for the inaccuracy of sequence-based methods for coreceptor prediction. On the other hand, we show how the location of the V3 loop sequence in sequence space can be used to improve the accuracy of the prediction of coreceptor usage. We further investigate the relation between the location of V3 loop sequences in sequence space and the associated clinical markers such as CD4^+^ T-cell level. Sequences of patients with a functioning immune system tend to be located close to each other in sequence space and thus are likely to share common features whereas, with decreasing CD4^+^ T-cell counts the conservation of the V3 loop among patients decreases and the diversity of possible viral genotypes increases. These results support the hypothesis of the immune system initially imposing strong selective pressure on the viral envelope gene. Once the immune system is compromised, this pressure diminishes which enables the virus to undergo less restrained variation.

## Materials and Methods

### Data preparation

Using the Los Alamos database [9] we defined two sets of labeled V3 loop protein sequences: the labels of the first set, which we call *NSI/SI* are attributed according to the annotation of non-syncytium-inducing (NSI) and syncytium-inducing (SI) strains. Those of the second set which we call *R5/X4* are attributed according to the annotation of the sequences concerning coreceptor usage – CCR5-, CXCR4- and dual-tropic (R5X4) strains. In order to prevent samples from a single patient to dominate any of the two sequence sets and to analyze viral evolution among hosts rather than patient-specific selection pressures, we limited our datasets to contain one randomly chosen sequence from each patient. The two sets contain 1096 and 859 V3 loop amino acid sequences, respectively, with an 85% prevalence of NSI and CCR5-tropic strains, respectively.

We used four sequence distance measures to compare the V3 sequences: Hamming distance, Blosum62 matrix [10], difference in amino acid charge and size, and difference in amino-acid composition at positions significant for the phenotype, as reported by Sing et al. [8]. We noticed that different distance measures result in the same pattern of sequence separation and therefore we decided to apply the Blosum62 matrix as the distance measure in other parts of this analysis.

### Sequence clustering

Both datasets were clustered hierarchically, using complete linkage clustering. We analyzed the tendency of viral sequences of different phenotypes to form clusters depending on the cluster diameter i.e. the distance between the two most distant elements of the cluster. Clustering with a given upper limit for the diameter was achieved in an iterative procedure of merging two closest clusters in each step of the procedure until no two clusters can be merged without generating clusters of a diameter above the predefined limit. In complete linkage clustering, the distance between two clusters is defined as the largest distance between two elements, one in each cluster. Only clusters containing at least 1% of all sequences in the dataset were considered, we define the sequences belonging to smaller clusters as well as singletons as *unclustered*. We additionally use a weighted notion of a cluster size such as to compensate for the imbalance between the amount of CCR5/NSI and CXCR4/SI sequences. The number of sequences of a given type in a cluster is multiplied by the ratio of the number of sequences of all other types in the full dataset over the number of sequences of the same type. This reweighting allows for considering smaller clusters of an underrepresented phenotype as significant.

The notion of silhouette value [11] was used to choose one clustering whose individual cluster structures should be investigated. The silhouette value of a sequence in a cluster is defined as the difference between the average distance of the sequence to sequences in other clusters and to sequences in the same cluster. The silhouette value of a cluster is the average silhouette value of its sequences. The silhouette value of a collection of clusters is the average of the silhouette values of its clusters. This measure can be used as a quantitative indicator of the coherence of a collection of clusters - larger values represent clusterings containing clearly separated, coherent clusters. We calculated the average silhouette values for clusterings obtained in successive steps of the hierarchical clustering and selected the clustering exhibiting a maximal silhouette value, among those clusterings that cluster more than 50% of the sequences in a dataset and contain more than one cluster. We call the clustering resulting from this procedure the *selected* clustering.

For analyzing the selected clustering we used an unsupervised learning method of data density estimation via classification [12]. This method allows for determining regions in sequence space in which the sequence density is significantly higher than average. For this purpose, we augmented the datasets with 500000 random reference data points distributed uniformly over the high-dimensional sequence space. The number of the reference data points was chosen as a balance between the computational load and sufficient space fill for accurate density estimation. A binary logistic regression model where true data points are assigned the value 1 and the generated reference data points have the value 0, was fitted using maximum likelihood estimation. The value returned by the fitted logistic regression for each of the true data points is treated as the probability of a point to be sampled by a distribution producing the analyzed dataset. The log-odds of this probability for each data point represent the local density of the original data relative to the generated reference data. Data density of a single cluster was calculated as the mean of log-odds of the cluster sequences. The larger the density of a cluster is, the more highly concentrated set of data points it contains.

We additionally measured the amount of positive selection among sequences in each cluster. For this purpose each of the protein sequences has been assigned its corresponding DNA sequence from the Los Alamos database. We define the cluster center to be the location of a sequence with minimal distance to all other sequences in the cluster. The amount of positive selection 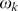 exerted on a sequence 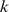 in a cluster is in terms of the mean of the ratio of non-synonymous to synonymous substitution rate 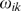 [13] of the given sequence and other sequences 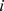 having a smaller distance 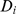 to the cluster center:
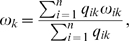
where 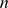 is the number of sequences in the cluster, 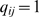 if 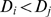 and 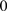 otherwise. Non-synonymous to synonymous substitution rates are calculated using the Yang and Nielsen method [13] implemented as a part of PAML software package [14].

### Phylogenetic analysis

The phylogenetic analysis was performed using the Splitstree software [15]. The split decomposition method [16] relaxes the usual requirement of representing the data in tree form, in order to elicit where the underlying distance matrix does not reflect a tree structure. A Splitstree network is tree-like, in general, but also represents the divergence of the phylogenetic data from the tree form by sets of parallel edges that expand the tree to a more complex network. The Blosum62 matrix was used as the distance measure in the Splitstree analysis.

### Accuracy of genotypic coreceptor prediction in sequence space

There is a range of computational methods that aim at distinguishing NSI/CCR5-only from SI/CXCR4-capable sequences based on the V3 loop sequence. We checked the accuracies of several sequence-based coreceptor prediction methods (11/25 rule, Web PSSM, geno2pheno) [6], [7], [8] on both V3 loop datasets. Sequences that are incorrectly classified by all considered methods were identified and localized in sequence space using the same sequence distance, clustering and phylogenetic analysis. We performed a test of the dependency of the accuracy of the predictions on the amount of data. In this test we used support vector machine (SVM) with linear kernel implemented in the package libsvm and position-specific scoring matrix (PSSM) implemented according to the description in [7].

In the search of possible reasons for errors in the V3 loop-based coreceptor prediction we analyzed an additional dataset of phenotyped sequence spanning both V2 and V3 regions. The dataset was retrieved from the Los Alamos database [9] and contains 280 sequences with 212 CCR5-, 34 CXCR4- and 34 dual-tropic. We compared the distribution of distances between sequences of the same and opposite tropisms of the full sequences and their V2 and V3 parts separately.

### Sequence space-based coreceptor prediction

The study described above – distance, clustering and phylogenetic analysis reveals a clear separation between NSI/CCR5-tropic and SI/CRCX4-tropic sequences in terms of the distance distribution, clustering steps and locations in splitstrees. NSI/CCR5-tropic sequences appear to be more conserved and to form clusters while SI/CXCR4 diverge in an apparently unconstrained manner and occupy distant parts of sequence space. Based on the above observations we tested if the position of a sequence relative to conserved NSI/CCR5-tropic V3 loop sequences in sequence space conveys sufficient information for the effective coreceptor prediction. We investigated the predictive power of the three aforementioned methods of characterizing sequence space based on distances, clustering and phylogeny on the NSI/SI and R5/X4 datasets separately. The proposed classification methods aim at distinguishing NSI/CCR5-only from SI/CXCR4-capable sequences. The score of each classifier is based on the separation of the sequence from the NSI/CCR5-tropic sequences in sequence space. Low scores characterize sequences less separated from NSI/CCR5-tropic sequences and therefore more conserved and probable to be NSI/CCR5-tropic. High scores indicate divergent sequences that, according to our sequence space analysis, are more likely to be SI/CXCR4-capable.

The classifiers were evaluated on both the NSI/SI and R5/X4 datasets using ten times ten-fold (10×10) cross-validation. In the following steps of the cross-validation procedure training and test sets are derived from the analyzed datasets.

The first classifier is based on the distance measures and predicts the tropism of a sequence depending on its average distance from all NSI/CCR5-tropic sequences in the training dataset. We constructed classifiers using three distance measures – Blosum62 matrix, Hamming distance and differences on positions significant for the coreceptor tropism according to Sing et al. [8]. As the coreceptor prediction score we use the mean distance of a sequence to all the NSI/CCR5-tropic sequences in the training set.

The second classifier predicts the coreceptor of a sequence according to the step of the hierarchical clustering algorithm in which the sequence ceases to be a singleton (called the clustering step for short). In order for the score to reflect the divergence of a sequence from the NSI/CCR5-tropic sequences, only these sequences from the training set are used in the prediction procedure.

The third classifier uses the Splitstree method for estimating the phylogenetic distance between pairs of sequences. Due to the high computational cost of a large splitstree construction, the phylogenetic distance between two sequences of a large dataset is calculated as an average distance between those two sequences in trees of randomly sampled sequence subsets composed in half of NSI/CCR5-only and in half of SI/CXCR4-capable sequences. The training procedure consists of 100 iterations of sequence sampling, tree construction and tree distances extraction. First, subsets of 100 sequences of the training dataset (50 NSI/CCR5-tropic and 50 SI/CXCR4-capable) are sampled. Then a splitstree is constructed for each of the sampled sets and for each sequence pair in the trees the information on phylogenetic distance between the two sequences is extracted. We tested the predictive power of two different measures of phylogenetic distance: the sum of the lengths of the splits separating two sequences and the number of splits between them. After the iterative sampling and tree construction procedure, additional trees are constructed containing the sequences in the training dataset that did not appear in any of the sampled trees. The distance between two sequences is the mean of distances between those two sequences in the trees in which both sequences appeared. This way a phylogenetic distance matrix of the training set is assembled. For the prediction step we select from the distance matrix a subset of 90 sequences: 45 NSI/CCR5-tropic that are most conserved (have the least number of splits or shortest splits separating them from other sequences) and 45 SI/CXCR4-tropic that are the most diverse (have the largest number of splits or longest splits separating them from other sequences). These sequences are used in the prediction procedure on the test set. In the prediction procedure subsets of 10 sequences from the test set are added to the selected 90 sequences of the training set and a splitstree is constructed for the merged set. The proportion of the test to train set sequences on this tree was chosen as the optimal after testing several other proportions for the accuracy of predictions. The mean number of splits and the mean sum of lengths of splits between a sequence and the NSI/CCR5-tropic sequences on the tree are used as the score predictive of the coreceptor usage.

All three classifiers were tested on both the NSI/SI and R5/X4 datasets using ten times ten-fold (10×10) cross-validation. We compared the performance of sequence space classifiers to three existing methods - SVM, PSSM and 11/25 rule. SVMs were trained using the package libsvm with linear kernel. PSSMs were implemented according to the description in [7]. All methods were evaluated using receiver operating characteristic (ROC) curves focusing on the trade-off between false positive (FPR) and true positive rates (TPR) which can be controlled by choosing a prediction cutoff for turning the continuous scores into actual class predictions. The area under ROC curve (AUC) was taken as a cutoff-independent class separation criterion. Averaged ROC curves were estimated from the 10×10 individual cross-validation curves using vertical averaging. In the analysis we used the ROCR package [17].

After comparing the performance of each of the proposed prediction methods individually, we additionally tested if adding the sequence space information to the SVM or combining several prediction methods into one can result in improved predictions. In the first approach we added to the binary feature vector coding the given sequence for the SVM the description of its location in sequence space. As the description of the location in sequence space we tested both the output score of the proposed classifiers, thus the separation of a sequence from the NSI/CCR5-tropic sequences, as well as the sequence and phylogenetic distance to each of the NSI/CCR5-tropic sequences. In the second approach we combined scores of several predictors, trained on the same training set, into one score. The scores of each predictor were normalized to the 0-1 interval with the higher scores representing CXCR4-capable sequences. We tested several methods of combining prediction scores, such as min, max, mean and Euclidian distance from the origin of the score space. We restricted the classifier combination methods to the simple, non-trainable combiners, bearing in mind their generally good performance [18].

### CD4^+^ T-cell counts in sequence space

In the last part of the study we related the location of a V3 loop sequence in sequence space to the corresponding patient CD4^+^ T-cell count. From the Los Alamos database we selected a set of 7003 V3 loop sequences with a reported CD4^+^ T-cell count. We additionally selected sequence samples and the corresponding CD4^+^ T-cell counts of 88 patients (225 sequences) from the University of Cologne. Since both Los Alamos and Cologne sequence sets exhibited similarities in the sequence space arrangement we merged them into a single dataset which we call the *full dataset*. For the purpose of the longitudinal study we allowed this dataset to contain more than one sequence of the same patient. From the full dataset we selected therapy-naïve patient samples (2213 sequences). We call this subset of the full dataset the *therapy-naïve dataset*. In both the full and the therapy-naïve datasets we distinguished longitudinal (time-series) data comprising sample sequences of the same patient spanning several years (72 patients in the full dataset, 16 therapy-naïve patients with an average of 3.1 and 3.9 sequences per patient respectively). We analyzed the longitudinal patient data in both datasets to see how sequences of viral variants inside an individual patient trace paths in sequence space in association with the progression of the disease. Finally, as in the initial part of this study (see Sequence clustering) we selected a clustering of the full and therapy-naïve sequence sets using the silhouette value and associated a single cluster sequence composition to the CD4^+^ T-cell counts.

## Results

### Sequence distance distribution

**Figure 1:**
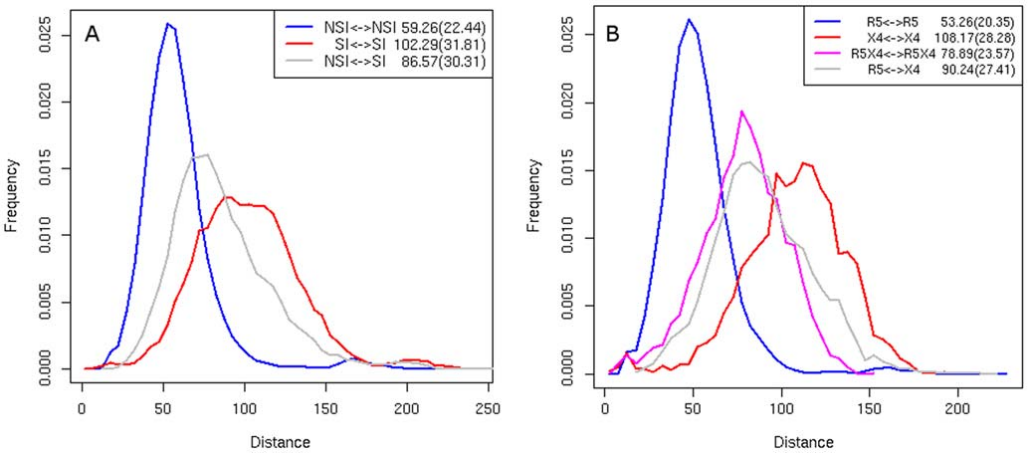
V3 loop sequence distance distribution. Shown is the distribution of Blosum62 distances between pairs of sequences of the same (SI vs SI, NSI vs NSI) and different (SI vs NSI) phenotypes (A) and of the same (CCR5 vs CCR5, CXCR4 vs CXCR4, R5X4 vs R5X4) and different (CCR5 vs CXCR4) tropisms (B). The mean value and standard deviation of each of the distributions are indicated in the inserted boxes.

For all considered sequence distance measures we observed the following pattern among the V3 loop sequences. CCR5-tropic and NSI sequences cluster strongly while, in contrast, CXCR4-tropic as well as SI sequences are much more widely spread out in sequence space. Figure 1A shows the distribution of Blosum62 distances between pairs of sequences from the NSI/SI dataset of the same and of different phenotypes. The mean distance of pairs of SI sequences (red curve) is almost twofold larger than the mean distance of the NSI sequences (blue curve). The distribution of the distances for pairs of SI sequences has also a larger variance than the one of the NSI sequences. The fact that the mean distance between pairs of sequences of opposite phenotypes is smaller than the mean distance of pairs of SI sequences implies that the SI sequences are widely spread out throughout sequence space and show no apparent common pattern of evolution. Distances between sequences in the R5/X4 dataset exhibit a similar pattern (Figure 1B) with the dual-tropic sequences spread out less, on average, than the CXCR4 sequences and more than the CCR5 sequences. The different distance measures result in the same pattern of sequence separation we therefore chose the Blosum62 matrix as the distance measure in all other parts of this analysis.

### Sequence clustering

**Figure 2:**
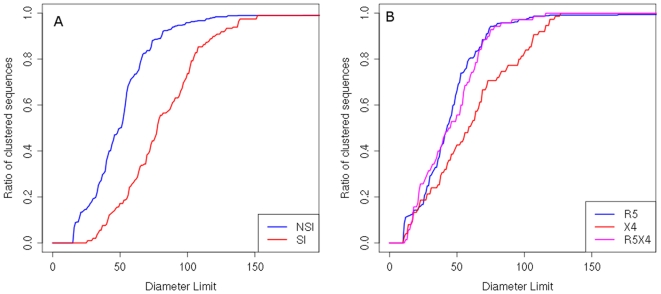
Clustering of the V3 loop sequences. The plot illustrates clustering trends of sequences of the NSI and SI phenotype (A) and of CCR5-, CXCR4- and dual-tropic sequences (B). The diameter limit, plotted on the x-axis, is defined as the distance between the two most distant elements of a cluster. The y-axis indicates the fraction of sequences in the dataset falling into any of the clusters below the diameter limit. Minimal cluster size is 1% of all sequences in the dataset, sequences of clusters of a smaller size as well as singletons are considered as unclustered and are not counted.

Clustering of the NSI/SI and R5/X4 datasets displays a more pronounced grouping trend of the NSI and CCR5-tropic sequences than of the SI and CXCR4-tropic sequences. In the initial steps of the iterative clustering procedure (see Sequence clustering part of the Materials and Methods section) only tight clusters of highly similar sequences are formed. In our dataset these clusters contain mainly CCR5-tropic and NSI sequences. The CXCR4/SI sequences are clustered only if we allow for clusters of a relatively large diameter – clusters of diameter large enough to contain 90% of the NSI sequences group only 50% of the SI sequences in the NSI/SI dataset (Figure 2A). The clustering of the R5/X4 dataset shows that dual-tropic sequences cluster relatively less than CCR5 but more than CXCR4 sequences – a clustering containing from 50% to 80% of the CCR5 sequences includes on average 10% less of the dual-tropic sequences (Figure 2B).

The selected clustering of both the NSI/SI and R5/X4 datasets was found according to the silhouette value described in the Materials and Methods and inspected in detail. In the NSI/SI dataset we obtained one major cluster containing 60% of all sequences, most of them of the NSI phenotype, and two smaller clusters each one containing 15% of all sequences, the first including solely NSI sequences, the second including equal percentage of sequences of both phenotypes. In the R5/X4 dataset the clustering contains two main clusters comprising 35% and 32% of all sequences respectively, containing mainly CCR5- and dual-tropic sequences, in the range of 19 to 38% of the sequences of each type in the R5/X4 dataset. Details of individual clusters are listed in Table 1. We observe that clusters containing mainly CXCR4/SI sequences are rare (only cluster 4 and 5 in the NSI/SI dataset); sequences of this phenotype tend to associate predominantly with CCR5 clusters. Additionally, in both datasets CXCR4/SI are highly over-represented among the unclustered sequences (p-value <0.001, chi-square test).

**Table 1:** Five largest clusters in the NSI/SI and R5/X4 dataset clustering.

dataset	NSI/SI			R5/X4			
cluster	all	NSI	SI	all	R5	X4	dual
1	0.59	0.65	0.33	0.35	0.38	0.09	0.30
2	0.15	0.17	0.06	0.32	0.37	0.05	0.19
3	0.14	0.14	0.15	0.06	0.07	0	0.04
4	0.02	0	0.07	0.05	0.06	0	0.01
5	0.01	0.01	0.03	0.05	0.06	0	0

Numbers indicate what fraction of the whole dataset is grouped in a given cluster (column “all”) and what is the ratio of the sequences of a given phenotype to all sequences in the respective cluster.

**Figure 3:**
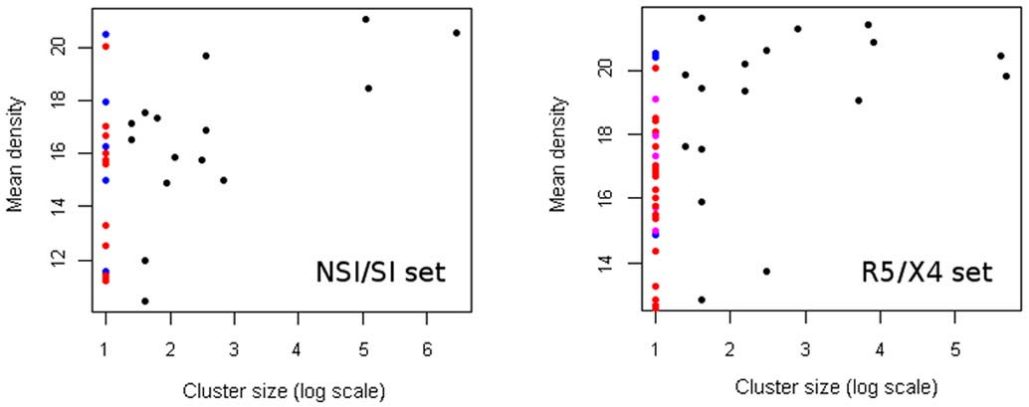
Data density in clusters of the selected clustering. Mean data density of sequences in clusters in the selected clustering of the NSI/SI (A) and R5/X4 dataset (B) is plotted against the cluster size. Single unclustered sequences are represented by dots at the value 1 on the x-axis with colors corresponding to their phenotype: NSI/CCR5 sequences in blue, SI/CXCR4 in red, R5X4 in magenta. Clusters are represented by black dots, cluster sizes are displayed in log scale. Large clusters are formed in denser parts of the data space than unclustered sequences. SI/CXCR4 sequences remain predominantly unclustered.

**Figure 4:**
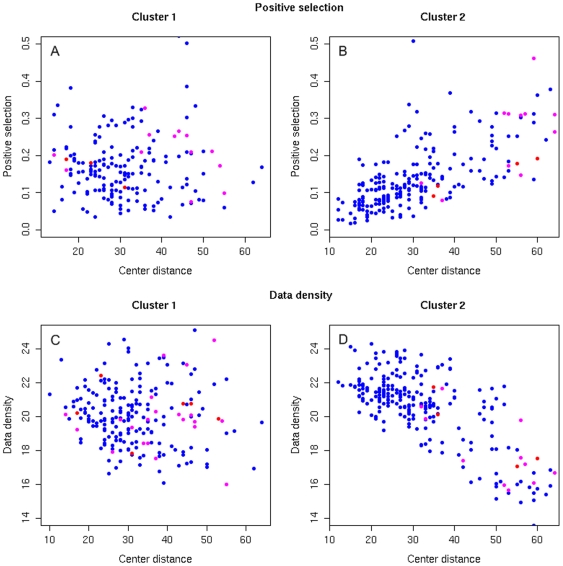
Structure of two largest clusters in the R5/X4 dataset. Cluster 2 shows a clear correlation of the distance of a sequence to the cluster center with the amount of positive selection on this sequence (diagram B) and with the data density of its sequences (diagram D). Dots on diagrams are colored according to sequence tropism (CCR5 - blue, CXCR4 – red, R5X4 - magenta). The plots indicate that cluster 2 has a dense center composed of similar, CCR5 sequences and sparser brims composed of CXCR4 and R5X4 sequences that are subject to weaker selection pressures. No such pattern can be observed in the case of cluster 1 (diagrams A and C) that seems not to have a coherent centre but group a medium density region of sequences of various tropisms.

Subsequently, we used the data density estimation method to examine the structure of individual clusters in the selected clustering. Data density is an indicator of how much more are the sequences concentrated in a given part of sequence space relative to the rest of the space. The relation of the cluster size and the sequence space density is illustrated in Figure 3. Unclustered sequences occur in less dense parts of sequence space and are predominantly SI/CXCR4-tropic. Detailed inspection of the individual cluster structure allows for relating data density and the amount of positive selection on a sequence to the position of a given sequence within its cluster. Positive selection is a measure of the amount of change in the amino acid sequence and reflects the rate of evolution of a sequence. The sequence position in a cluster is characterized in terms of its distance from the cluster center. The two largest clusters of the R5/X4 dataset are depicted in Figure 4. In the case of cluster 2 we observe a strong correlation of the sequence distance from the cluster centre with the data density at the location of the sequence as well as with the amount of positive selection exerted on the sequence (correlation coefficient of -0.76 and 0.71, respectively). The results reveal that there is a dense center in cluster 2 grouping most of the cluster sequences and sparse brims where the concentration of sequences is smaller. The selection pressure in the center of such cluster is greater and reduced farther from its center. CXCR4- and dual-tropic sequences preferentially occupy the outer regions of the cluster (Figure 4). However, no such cluster pattern is observed in the case of cluster 1. Data density analysis shows that this cluster groups sequences spread over a similar density range independently of their position within the cluster. There is no clear distribution of variation in selection pressures in cluster 1 either.

### Phylogenetic analysis

**Figure 5:**
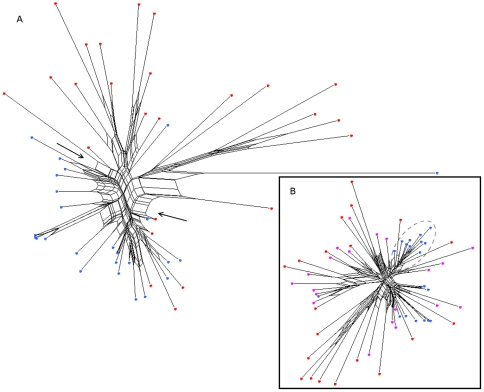
Splitstrees of sampled subsets of the NSI/SI and R5/X4 datasets. (A) The splitstree was generated on a randomly sampled set of 25 sequences of each phenotype. NSI sequences are represented by blue dots, SI by red dots. (B) The splitstree was generated for a randomly sampled set of 20 sequences of each tropism (CCR5 - blue dots, CXCR4 -red dots, R5X4 – magenta dots). Branch lengths, the number and width of splits (set of parallel edges in the graph) illustrate the evolutionary distance between the parts of the tree that they separate. An example split is indicated by two arrows in panel A. In both cases the SI/CXCR4 sequences are located on branches relatively longer than those of the NSI/CCR5 sequences and separated by wide splits from NSI/CCR5 phenotype (e. g. indicated by arrows). NSI/CCR5 sequences tend to form dense tree-like parts of the Splitstree network containing few short splits (example shown in dashed circle in panel B) which indicates their evolutionary proximity.

The visualization of the V3 loop data via the Splitstree diagrams illustrates the separation of viral strains of different coreceptor usage. Since both datasets are too large to be displayed by a single diagram, the diagrams are generated on randomly sampled subsets of sequences. Figure 5A shows an example Splitstree of a randomly sampled set of 25 sequences of each phenotype in the NSI/SI dataset. Both the lengths of splits (sets of parallel edges in the centre of the graph) and of single branches connecting data nodes to the rest of the tree clearly discriminate between these two types of sequences. SI sequences (represented by red dots in Figure 5A) are located on long tree branches that reflect the larger evolutionary distance between them and other sequences in the dataset. NSI sequences (blue dots) are located on shorter branches and grouped in more tree-like clades. A similar tree generated for a sample of 20 sequences of each tropism from the R5/X4 dataset is shown in Figure 5B. The dual-tropic sequences (represented by magenta dots) have an intermediate character between the CCR5- (blue dots) and CXCR4-tropic (red dots) sequences. Both their branch lengths and localization on the tree support the view of the dual-tropic sequences combining characteristics of the two other sequence types or being an intermediate form in their evolution. A test consisting of generating random trees of sequences in both datasets shows that in the R5/X4 dataset the average path joining two CXCR4-tropic sequences on a tree is about 1.35 times longer than the one joining two CCR5-tropic sequences. In the NSI/SI dataset an average path joining two SI sequences is 1.1 times longer that the one joining two NSI sequences.

The above analysis indicates that both CCR5-tropic and NSI sequences share common features and form coherent groups in sequence space. In the selected clustering 99% and 98% of the NSI and CCR5-tropic sequences from each dataset, respectively, are clustered. In the following part of this study, we therefore used these sequences as reference points in sequence space. The mean distance of a sequence from all CCR5/NSI sequences was considered as a measure of its conservation.

### Accuracy of genotypic coreceptor prediction in sequence space

**Figure 6:**
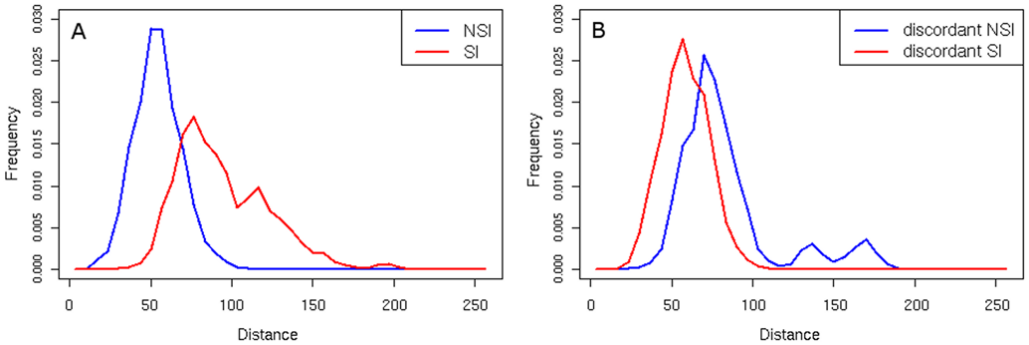
Distance distribution of incorrectly predicted V3 loop sequences. The plot contains the distribution of distances of the average distance of correctly (A) and incorrectly (B) predicted sequences to the set of NSI sequences with a phenotype correctly predicted by all analyzed coreceptor prediction methods (11/25 rule, Web PSSM, geno2pheno). NSI sequences are represented by the blue curve, SI by the red curve.

Even though the distance distributions as well as the Splitstree diagrams exhibited discernable differences between sequences with different phenotypes, still some exceptions could be observed. We examined the performance of common coreceptor prediction tools (11/25 rule, WebPSSM, geno2pheno) [6], [7], [8] on the NSI/SI and R5/X4 datasets and localized the incorrectly predicted sequences in sequence space. We measured the average distance of sequences in the NSI/SI dataset to a reference set composed of NSI sequences from this dataset with a phenotype correctly predicted by all three methods. Figure 6 shows the distribution of the average distance of four different groups of sequences to this reference set. The four groups of sequences are: (i) correctly classified NSI sequences (A, blue curve), (ii) correctly classified SI sequences (A, red curve), (iii) NSI sequences misclassified by all three methods (B, blue curve) and SI sequences misclassified by all three methods (iv) (B, red curve). Sequences that fail to be correctly classified are located in untypical regions in sequence space – SI sequences classified in discordance with the Los Alamos annotation are closer to correctly predicted NSI sequences than the correctly predicted SI sequences (p-value <0.05) and, on the other hand, the misclassified NSI sequences are further apart from this reference set in sequence space than the correctly predicted NSI sequences (p-value <0.05). A similar significant pattern can be observed for the R5/X4 dataset.

**Figure 7:**
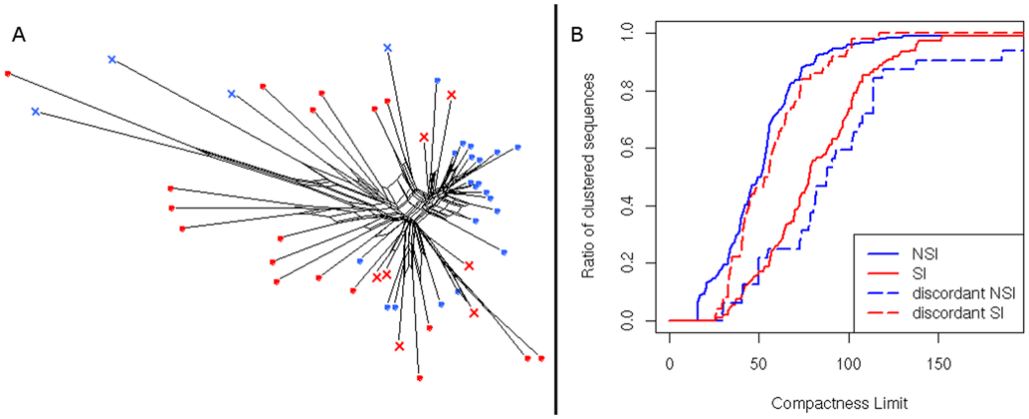
Location of the incorrectly predicted sequences in sequence space. (A) The splitstree was generated for a sample of sequences containing misclassified NSI sequences represented by blue crosses and misclassified SI nodes by red crosses. Correctly classified sequences are represented by dots, colors are in accordance to the coloring scheme in Figure 3. (B) Clustering patterns of sequences misclassified (dashed curves) by geno2pheno are plotted against to those correctly classified (solid curves).

The above observations were confirmed by the Splitstree analysis (Figure 7A). The misclassified sequences show evolutionary relationships characteristic for the opposite phenotype – NSI sequences occupy longer branches and are located among SI sequences on the tree while the misclassified SI sequences are evolutionarily less distant from NSI clades or lie on boundaries between both phenotypes. Figure 7B illustrates the clustering patterns of sequences misclassified by geno2pheno as compared to those correctly classified. As observed in the previous analysis the misclassified sequences show clustering trends uncommon for their phenotype.

To investigate whether the classification error is due to data scarceness, we examined the performance of two classification methods trained on datasets with various sizes. We tested the classification accuracy of support vector machine (SVM) and position specific score matrix (PSSM) – the computational methods used by geno2pheno and WebPSSM respectively. We sampled subsets of the original NSI/SI dataset, used them as training sets for the SVM and PSSM, and then verified the number of prediction errors of the trained model on the same sequence set. With the increasing size of the training and test dataset we could observe no tendency of a decreasing prediction error. Both methods failed on a similar percentage of sequences independently of the size of the underlying dataset.

**Figure 8:**
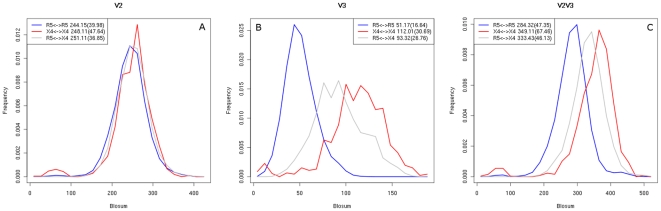
Distance distribution of V2-V3 sequences. Shown is the distribution of Blosum62 distances between pairs of V2 (A), V3 (B) and V2V3 (C) parts of sequences of the same (CCR5 vs CCR5, CXCR4 vs CXCR4) and different (CCR5 vs CXCR4) tropisms. The mean value and standard deviation of each of the distributions are indicated in the inserted boxes.

On the one hand, a possible reason for the errors of the coreceptor prediction tools might be the complexity of factors determining coreceptor usage. On the other hand, other parts of the gp120 protein beside the V3 loop may play a role in coreceptor attachment. We repeated a similar study on the V2 loop. However, the results showed no clear separation of the two phenotypes in sequence space. Diagrams in Figure 8 illustrate the distance distribution among the sequences spanning over both V2 and V3 regions as well as between their V2 and V3 parts separately. V2 sequences do not show the same pattern of distribution with highly divergent CXCR4-tropic and more conserved CCR5-tropic sequences as the V3 sequences show. The joint V2 and V3 regions have a lower difference in distance distribution between both sequence types than their V3 part separately.

### Sequence space-based coreceptor prediction

Based on the observations gathered from the sequence space analysis we suggested sequence space-based coreceptor prediction methods. The predictors are derived from a description of the localization of a sequence in sequence space in terms of either distance measures, phylogenetic distance estimated from splitstrees or clustering step. We compared the predictive performance of the proposed classifiers with the existing methods such as SVM, PSSM and 11/25 rule. The comparison has been done in the framework of ROC analysis [17] in order to analyze the tradeoff between true positive rate (TPR) and false positive rate (FPR) across the range of all possible cutoffs. The ROC curve is a plot of TPR and FPR when varying the score cutoff for classification over all possible values. We additionally use the size of the area under ROC curve as a cutoff-independent quality measure of classification.

**Figure 9:**
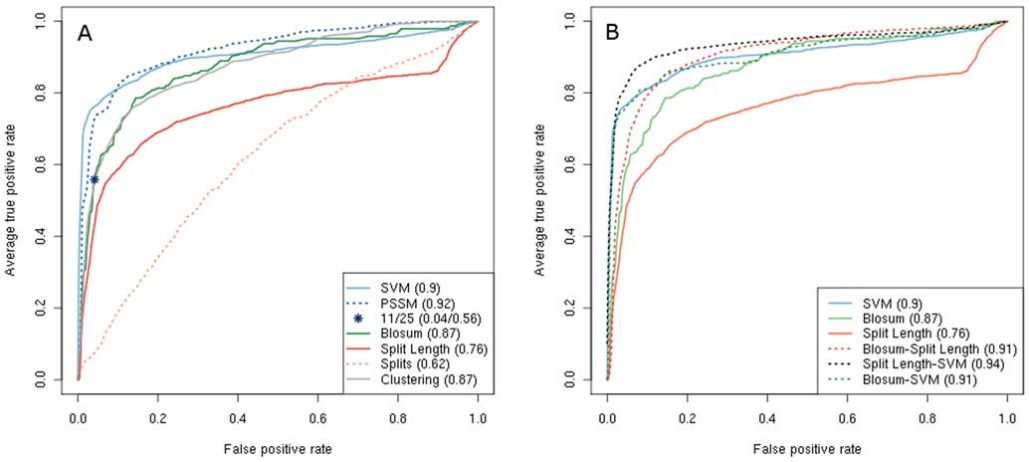
Performance of sequence space-based coreceptor prediction methods. Performance of the individual coreceptor prediction methods (A) and their selected combinations (B) on the R5/X4 dataset is illustrated by the ROC curves. The AUC of each method is indicated in the inserted box.

All distance-based classifiers exhibit similar performance. At the FPR of the 11/25 rule (0.05 in the NSI/SI and 0.04 in the R5/X4 dataset) the distance methods have the TPR between 0.51 and 0.56 in both the NSI/SI and the R5/X4 dataset. The areas under the ROC curve (AUC) reach from 0.85 to 0.88 in both datasets. This performance is slightly worse than SVM and PSSM methods that show TPR between 0.71 and 0.76 at the FPR of 11/25 rule and the AUC of about 0.90 and 0.92 (SVM and PSSM respectively) in both datasets (Figure 9A).

The cluster-based classifier performs similarly to the distance based ones with the TPR of 0.54 and 0.53 and AUC 0.86 and 0.87 in the NSI/SI and R5/X4 datasets respectively.

The classifier based on the number of splits separating a sequence from the CCR5-only class of sequences performs significantly worse than the distance-based methods. It yields an AUC of 0.65 and 0.62 in the NSI/SI and R5/X4 dataset, respectively, and a TPR of 0.19 and 0.07 respectively at the FPR of the 11/25 rule. This suggests that similar ranges of split numbers can separate the sequences of both classes from the conserved CCR5 class of sequences. A better prediction performance is achieved with the use of the sum of lengths of splits that separate a sequence from these CCR5 sequences – TPR of 0.47 and 0.45 in NSI/SI and R5/X4 datasets respectively with the AUC of 0.74 and 0.76 respectively. However, this result is still much lower then other sequence space-based methods.

No sequence space-based methods achieved the performance level of the commonly used methods such as SVM and PSSM (see Figure 9A). However, we tested if combining different prediction methods could improve the results. First, we used the description of the position of a sequence in sequence space, either in terms of the score of each of the sequence space prediction methods or in terms of a vector of distances to each NSI/CCR5-tropic sequence, and added it to the binary feature vector coding the given sequence for the SVM. However, none of the resulting enriched SVMs showed better performance than the one based on the sequence only.

Next, we combined the scores of several predictors into a single score. We started by comparing the scores returned by different classification methods within the same cross-validation run. All the methods show a high correlation in the scoring (Pearson correlation coefficient >0.8) with the exception of phylogeny-based and SVM classifiers (correlation of about 0.25 and 0.7, respectively). Despite the low prediction power of the phylogeny-based method, we observed several sequences (about 15 in both datasets in each cross-validation run) where the score returned by this classifier is 50% more accurate than the score of the SVM. However, since the phylogeny-based classifier contains a stochastic step, the scores returned by this method have a high variation between different cross-validation runs and this result is not reproducible in each run. We then tested several methods for combining scores and found the Euclidian distance from the origin of the score space to be the combiner achieving the best discrimination between the two sequence classes. Finally, we examined all possible subsets of predictors and found that combining classifiers improves their performance in general. Three distance-based classifiers coupled together achieve better results than each one individually (Table 2). Joining the phylogeny-based method with another classifier improves the predictive power the most - up to 0.03 increase in the AUC in the case of SVM (Figure 9B). The largest predictive power was achieved by merging the distance and phylogeny scores with the SVM methods (Table 2).

**Table 2:** Performance of coreceptor prediction methods and their combinations on the NSI/SI and R5/X4 datasets.

	R5/X4		NSI/SI	
Predictor	TPR at 0.04 FPR	AUC	TPR at 0.05 FPR	AUC
**Blosum**	0.5544	0.8747	0.5606	0.8647
**Hamming**	0.5241	0.8842	0.5144	0.8644
**Significant Positions**	0.5524	0.8765	0.5499	0.8532
**Split Number**	0.0712	0.6245	0.1932	0.6486
**Split Length**	0.4262	0.7591	0.4722	0.7449
**Clustering**	0.5510	0.8725	0.5399	0.8606
**SVM**	0.7607	0.9038	0.7407	0.8887
**PSSM**	0.7276	0.9199	0.7140	0.9131
**Blosum-Hamming-Significant Positions**	0.6014	0.8990	0.5808	0.8745
**Blosum-Split Lengths**	0.5986	0.9063	0.6076	0.8768
**Blosum-SVM**	0.7517	0.9062	0.7313	0.8944
**Split Length-SVM**	0.8062	0.9355	0.7929	0.9156
**Blosum-Split Length-SVM**	0.8076	0.9420	0.7778	0.9224
**All Methods**	0.7607	0.9404	0.7369	0.9178

The performance of the analyzed prediction methods is assessed with the TPR at the FPR of the 11/25 rule (0.05 in the NSI/SI and 0.04 in the R5/X4 dataset) and with a cutoff-independent measure of the size of the area under ROC curve. The table lists the performances of several classification methods and their combinations. The predictions of the combined methods are calculated as the Euclidian distance from the origin of the prediction score space of each of the individual methods.

### CD4^+^ T-cell counts in sequence space

**Figure 10:**
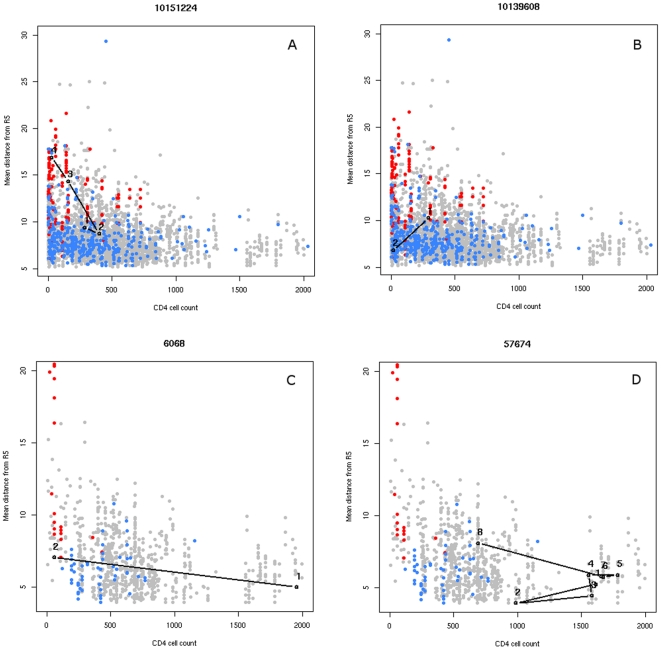
Patient sequence evolution in sequence space. CD4+ cell count level is plotted against mean distance to NSI/CCR5 sequence set in the full (A and B) and the therapy-naïve datasets (C and D). Sequences with annotated phenotype or tropism are marked with colors (blue for NSI/CCR5 sequences, red for SI/CXCR4). Sequential data points of the same patients are marked in black and connected with a solid line: (A) therapy-experienced patient showing an increase in sequence variability with decreasing CD4+ cell count, (B) therapy-experienced patient with an opposite trend, (C) therapy-naïve patient showing an increase in sequence variability with decreasing CD4+ cell count, (D) therapy-naive patient with a mixed trend. Patient identifiers are indicated above the plots.

In the last part of this study we aimed at relating the position of V3 loop sequences in sequence space to the CD4^+^ T-cell count. We performed this part of the analysis on the full dataset – the set of sequences with a reported CD4^+^ T-cell count. As in the analysis of misclassified sequences in the NSI/SI and R5/X4 datasets, we used the mean distance to the sequences annotated as NSI or CCR5-tropic in the full dataset (653 sequences) as a measure of sequence conservation. For each sequence in the full dataset, we calculated this distance and plotted it against the CD4^+^ T-cell count (Figure 10A and B). Among sequences collected from highly immunosuppressed patients (T-cell count below 200 cells/mm^3^) we observe a large range of sequence conservation spanning conserved, NSI/CCR5-like, as well as highly divergent sequences. Among the patients with higher T-cell counts this range is narrower and includes only conserved sequences.

We next analyzed the longitudinal patient data in the full datasets to see how divergence of viral variants inside an individual patient can change in association with the disease progression. In the full dataset we counted 76 patients (both therapy-naïve and therapy-experienced) with data occupying several time points. We examined the conservation of sequential measurements of each of the selected patients against the corresponding CD4^+^ T-cell count. For 21 patients we could observe an increase in sequence divergence with decreasing CD4^+^ T-cell count to the immunodeficiency level - below 200cells/mm^3^ (example in Figure 10A). Five patients show an opposite trend with a decrease in mutations co-occurring with a decrease of the number of CD4^+^ T-cells (see example in Figure 10B). Remaining patients showed no or various conservation change with varying CD4^+^ T-cell count level.

In order to see how drug therapies can influence the relationship between sequence conservation and CD4^+^ T-cell levels, we performed the same study on the data of untreated patients included in the therapy-naïve dataset. This dataset contains 2213 sequences, 112 of which are annotated as NSI or CCR5-tropic. Other sequences are annotated as SI/CXCR4-tropic or have no annotation. The NSI/CCR5 sequences were used as a reference set in the sequence space. In the therapy-naïve dataset the lack of conservation of the sequences of patients with an impaired immune system is more pronounced. Among severely immunosuppressed patients (CD4^+^ T-cell count below 200 cells/mm^3^), almost no highly conserved sequences can be observed (Figure 10C and D). Again, patients with higher T-cell counts do not exhibit highly divergent viral strains.

The longitudinal data analysis revealed that among 16 patients with more than one time point measurement, four show an increase in the sequence divergence with the transition to the immunodeficiency state (T-cell count below 200 cells/mm^3^) (example in Figure 10C). However, none of the patients shows the opposite trend.

As in the previous part of this study, we used the silhouette value to determine the selected clustering of the full and the therapy-naïve datasets. Both sequence sets contain one major cluster containing 86% and 52% of all sequences, respectively, and three smaller clusters of about 3% and 5% in each set respectively. We observed an important overrepresentation of samples collected from ill patients (T-cell count below 500 cells/mm^3^) among the unclustered sequences (p<0.001, chi-square test) which reflects their high evolutionary divergence.

## Discussion

By means of different distance measures, clustering and phylogenetic methods, the present study illustrates and interprets patterns in V3 loop sequence space of the HIV-1 envelope gene. The analysis confirms a relatively high conservation of CCR5-tropic and NSI viral sequences as compared to more highly divergent CXCR4-tropic and SI sequences. According to our study, the CCR5/NSI sequences appear to share common features and the CXCR4/SI sequences to be highly divergent and not showing a unique mutation pattern. Other studies detect at least several possible V3 loop mutation pathways [19] and indicate the twofold larger heterogeneity of the X4-tropic viruses over the R5-tropic. The lack of common features among the CXCR4 sequences, as well as their high divergence, render these sequences impossible to group in coherent clusters. A statistical model of CXCR4 sequences is therefore difficult to obtain. This divergence pattern has already been reported in previous studies [20], [21], [22], in contrast to these analyses done on small data samples, we support these hypotheses with a large-scale analysis of V3 loop sequence data.

The analysis of cluster structure revealed the existence of dense regions of sequence space occupied by CCR5-tropic sequences sparsely surrounded and interspersed by CRCX4- and dual-tropic sequences. The sparse outer boundaries of these regions are less highly conserved and are under positive selection. The finding of such regions suggests that the coreceptor usage switch which is correlated with high sequence divergence might be driven by the lack of selective pressure on the viral V3 loop. This finding supports other studies that report presence of selective pressure maintaining the relative homogeneity of the CCR5 viruses as well as the correlation of the emergence of the CXCR4-tropic strains with the accelerated V3 loop [20], [23].

Localization of the misclassified sequences in sequence space revealed a possible reason for errors of V3 loop sequence-based coreceptor prediction tools. These sequences are located in parts of the sequence space untypical for their phenotype and show an inverse pattern in their distances distribution as compared to the distances among correctly predicted sequences (Figure 6). Only the sequences misclassified by the 11/25 rule do not exhibit such a distance inversion (data not shown) which suggests that in certain cases mutations at positions 11 and 25 are insufficient for the change of phenotype and that the accumulation of other mutations on the V3 loop might drive the coreceptor switch. Previous studies [24] report dual-tropic V3 loop sequences as being predominant among the sequences misclassified by different prediction methods. It may be that the sequences we observe to be located in untypical regions of sequence space for their tropism represent an intermediate form between the two mono-tropic types. Other studies [25] reveal a dependence of the predictive value of positions 11 and 25 on CD4^+^ T-cell level, suggesting that individual patient parameters can influence the viral coreceptor usage. Another possible reason for errors in predicting coreceptor usage on the basis of V3 sequence may be the occurrence of complementary mutations in other parts of the gp120 protein. However, a similar inspection of the sequence space of the V2-V3 sequences revealed no separation between the CCR5 and CXCR4 phenotypes in the V2 part (Figure 8). A similar observation has been previously reported [26] in an analysis of the V1-C3 region of the gp120 protein sequences that revealed a relative conservation of the V2 region with only a few positions in the V1/V2 stem being significant for the coreceptor usage. This strongly points to the V3 loop being the region of the gp120 protein crucial for the viral tropism that is under selection pressures driven by the interaction with the host.

The separation of the two classes of sequences in sequence space can therefore be used for the coreceptor usage prediction. Our prediction methods are drawn from sequence space localization determined by the means of sequence distance measures, phylogenetic distance and clustering. The predictive power of the methods is below that of SVM and PSSM which is not surprising as the prediction score based on sequence space is obtained by averaging over many sequence distances, an operation in which information on a single position in a sequence is lost. Adding the sequence space location descriptor to the sequence-based SVM did not improve its accuracy which might be due to the fact that the sequence space location is drawn from the genetic information that is already used by the SVM. However combining prediction methods, in particular the phylogeny-based method with other classifiers, resulted in a performance increase. Nevertheless the stochastic step involved in the phylogeny-based method renders its predictions less reliable which is reflected by the weak predictive power of the method by itself.

Relating clinical markers to the sequence space position shows higher sequence variability among patients with an impaired immune system. Other studies have reported the emergence of highly mutated viruses in the later stage of infection [23], [27]. Our analysis shows the association of this emergence with the drop in patient CD4^+^ T-cell count. This association might be due to a selection pressure exerted on the viral V3 loop that disappears with the gradual erosion of the immune system. With the attenuation of this selection force the virus is apparently undergoing an unrestricted evolution on the V3 loop which traverses distant parts of sequence space. The existence of a similar selection mechanism has been suggested in other studies [28].

However the nature of the selection pressure limiting the viral mutation in the early stage of infection is not clear. The observation that the development of a highly mutated CXCR4-tropic virus is associated with low CD4^+^ T-cell numbers and therefore with the impairment of the immune system suggests an immunological component of this mechanism. There is some evidence for selective pressure against the emergence of CXCR4 strains having an immunological basis [29] and decrease of positive selection accompanying the drop in CD4^+^ T-cell count. This supports the hypothesis that the emergence of mutated strains late in infection is related to the limitation in the suppressive capacity of the immune system. However, recent studies [30] show examples of patients exhibiting CXCR4-tropic virus at relatively high CD4^+^ T-cell counts. Such cases could be explained by a successful antiretroviral treatment, as viral tropism appears not to impact the response of CD4^+^ T-cell count on the treatment [31]. In our datasets we find patients (mostly therapy-experienced) who exhibit an increase in sequence conservation with a reduction of the CD4^+^ T-cell counts (example in Figure 10B). Other parameters may therefore influence the evolution of the viral V3 loop. Notably, high sequence conservation has been observed among patients under long-term successful therapy [32].

Our large-scale analysis of the sequence space of the V3 loop provides a comprehensive description of CCR5- and CXCR4-tropic viral phenotypes. By characterizing CXCR4 viruses as highly variable and dispersed in sequence space we provide further evidence for the fact that not only is this phenotypic change predictive of disease progression but also that it comes as a result of an extensive evolution of the V3 loop sequence and a decrease of the selective pressure on the viral envelope genome.
